# A Prospective, Randomised Controlled Trial Comparing the use of the Proximal Femoral Nail – Antirotation and Dynamic Hip Screw for Stable Intertrochanteric Femur Fractures-Stable Trochanteric Fractures Intramedullary versus Extramedullary (STRIVE) Study

**DOI:** 10.5704/MOJ.2503.011

**Published:** 2025-03

**Authors:** QY Yeo, KRP Pillay, M Tan, THI Chua, BKE Kwek

**Affiliations:** 1 Department of Orthopaedic Surgery, Woodlands Health Campus, Singapore; 2 Department of Orthopaedic Surgery, Tan Tock Seng Hospital, Singapore

**Keywords:** stable intertrochanteric hip fracture, proximal femoral nail anti-rotation (PFNA), dynamic hip screw (DHS)

## Abstract

**Introduction:**

Intramedullary nailing in the management of hip fractures is gaining in popularity. Our study aims to determine if there are any clinical and radiological differences between the Proximal Femoral Nail Antirotation II (PFNA II) and the Dynamic Hip Screw (DHS) in the management of stable intertrochanteric (IT) femur fractures. **Materials and methods:** This is a single blinded prospective randomised controlled trial of 33 patients, aged above 60, comparing the use of the PFNA II and the DHS for the treatment of stable IT femur fractures in a single tertiary centre with an established ortho-geriatric co-managed hip fracture care pathway.

**Results:**

Of the 33 patients enrolled, 18 patients were treated with the DHS and the rest with the PFNA II. The two groups had similar demographic profiles and pre-operative radiological parameters. There was no statistical difference between the two groups in terms of intra-operative bleeding, post-operative pain score and total surgical time. The median Harris Hip and Parker Mobility Scores for the DHS group were non-inferior compared to the PFNA II group. Surgical time, blood loss, post-op radiological parameters and functional outcomes including time to ambulation were similar in both groups.

**Conclusion:**

We recommend the use of the DHS for stable IT fracture patterns in view of its cost savings and equivalent functional and radiological outcomes.

## Introduction

Intertrochanteric (IT) hip fractures are the second most common fractures of the hip, associated with significant morbidity and mortality^1^. The preferred management for these fractures is surgical intervention with the aim to restore patients’ premorbid function and avoid complications secondary to immobilisation.

Surgical treatment options comprise of extramedullary and intramedullary devices. The Dynamic Hip Screw DHS, an extramedullary device, was introduced in the 1950s, and is still recognised as the standard device for fixation of intertrochanteric fractures^2,3^.

The Proximal Femoral Nail Antirotation II [PFNA II; Synthes GmbH, Oberdorf, Switzerland] is a commonly used intramedullary implant for the treatment of intertrochanteric fractures. It is inserted using a minimally invasive technique and designed for superior purchase of the femoral head by medial compaction of the cancellous bone, thereby improving rotational and angular stability in osteoporotic bone^3^.

The debate on the use of intramedullary nails (IM) in stable and unstable intertrochanteric fractures is still ongoing. Zehir *et al* compared the use of the PFNA with the DHS in unstable IT fractures in a prospective randomised study^4^. They found that the PFNA group had superior functional recovery, shorter operative time and less blood loss. In a four year follow-up study by Yu *et al*, the DHS had an increased risk for re-operation when compared with the PFNA in stable IT fractures^5^. In another study by Sharma *et al* similar functional outcomes between the two implants were seen with more technical errors seen with the use of the PFNA^6^.

We are seeing the use of the DHS dwindling as the popularity of the intramedullary nail rises. However, is this cheaper implant really obsolete in the fixation of IT fractures? In the current environment, where surgical technique for intramedullary nailing has been refined, are technical errors still an issue?

With a growing ageing population, the number of intertrochanteric fractures and invariably the number of stable intertrochanteric fractures is on an increasing trend. If there are no significant differences between the implants, then wouldn’t a cheaper option be better both from a patient’s and health economics perspective?

In view of the conflicting evidence guiding the optimal implant choice for fixation of stable IT fractures, we conducted a prospective study to compare the PFNA II and the DHS in the treatment of stable IT fractures, specifically evaluating fracture reduction, functional scores, and complications.

## Materials and Methods

This is a prospective, randomised controlled trial comparing the use of the PFNA II and the DHS for the treatment of stable IT femur fractures in a geriatric patient group. The study was approved by the ethical review board of the study institution and conducted in accordance with CONSORT (Consolidation Standards of Reporting Trials) 2010 guidelines. The patients were recruited from a single tertiary centre with an established ortho-geriatric co-managed hip fracture care pathway between June 2014 and December 2018.

We chose to focus on the geriatric patient age group above the age of 60 because these patients are more likely to sustain an intertrochanteric fracture and are more likely to have osteoporotic bone. Therefore, the results would give a better idea of comparison of the two implants in poorer quality bone and would improve the homogeneity of the study population.

Eligible patients were prospectively enrolled when they presented to the hospital with a stable hip fracture based on the following inclusion criteria: (i) age above 60 years, (ii) isolated stable closed IT fracture, (iii) definitive primary treatment with either the DHS or the PFNA II planned within 7 days of injury, and (iv) willing and able to comply with post-operative management program and follow-up. Stable IT hip fractures were defined by the Muller AO Classification (31-A1.1, 31-A1.2, 31-A1.3 or 31-A2.1)^7^.

The exclusion criteria were (i) pathological fractures, (ii) open fractures, (iii) polytrauma, (iv) active malignancy, (v) American Society of Anaesthesiologists (ASA) Classification V or VI, (vi) patients with limited life expectancy or were deemed surgically unfit due to significant medical comorbidities, (vii) neurological and psychiatric disorder that precluded reliable assessment (including dementia), (viii) drug or alcohol abuse, (ix) patients with known allergy to any component of the device, and (x) inability of the patient to walk prior to injury.

This was a single blinded study where the outcome assessor was blinded. Randomisation was via block randomisation. The randomisation codes were computer generated using a 1:1 ratio, in blocks of 6. Upon final confirmation of suitability with intra-operative post reduction fluoroscopic imaging, the sealed envelope with the randomisation code was opened by the performing surgeon and the patient was assigned a treatment, either the PFNA II or the DHS group. Both implants were on standby and made available at the time of surgery.

All surgeries were conducted or directly supervised by a fellowship trained orthopaedic trauma surgeon. They were performed under regional or general anaesthesia with preoperative antibiotic prophylaxis. Tranexamic acid was not administered to our study population. Closed reduction was attempted with the aid of a traction table.

For patients who had a DHS inserted, this was performed using a lateral approach. The tensor fascia lata was incised and vastus lateralis reflected anteriorly to expose the proximal femoral shaft. With the aid of a 135° Centrum-Column-Diaphysis (CCD) femoral jig, a guidewire was inserted to the required depth in the femoral head under fluoroscopic guidance. The screw length was measured before the triple reamer was advanced to the appropriate depth. Subsequently, the DHS was inserted until a satisfactory position was confirmed under intra-operative fluoroscopy. The length of the side plate and the use of a surgical drain were dependent on surgeon preference. No trochanteric side plate attachments were used as these were stable fracture patterns.

Patients who were randomised to the PFNA II had a 200mm PFNA II implant with 135° CCD inserted. An incision proximal to the tip of the greater trochanter was made and the guidewire was inserted. Proximal reaming was performed over the guidewire, the femoral canal was sized, and the appropriate nail inserted. A lateral stab incision was then made, and the guidewire for the PFNA II blade was inserted into the femoral head using the aiming arm. Reaming was performed over the guidewire and the blade was advanced into the femoral head to the appropriate depth. One distal locking screw was then inserted via the aiming arm. Use of an end cap or surgical drain were determined by the operating surgeon.

Surgical details such as type of anaesthesia, implant used, surgical time, estimated blood loss, intra-operative transfusion and usage of surgical drain were collated. Intra-operative complications such as loss of reduction, poor intra-operative fracture reduction, problems with guidewires, iatrogenic fractures, bleeding complications and change of surgical procedures were also tracked.

All patients in both groups underwent a standardised postoperative rehabilitation program that included standard surgical wound care, immediate full weight bearing ambulation and progressive strengthening exercises when the implant was noted to be stable based on radiological parameters. All patients received perioperative physiotherapist and occupational therapist assessments. The duration of acute hospitalisation stay was also recorded.

The patients were followed-up for a period of 12 months with serial clinical and radiological evaluations at 6 weeks, 12 weeks, 6 months and 1-year intervals. Clinical data collected included clinical assessments, wound complications, thromboembolic events, general medical complications (pneumonia, sepsis and death) and revision surgery. Functional scoring assessments using the Parker Mobility Score and the Harris Hip Score were gathered for all patients pre-operatively and throughout their follow-up.

The Parker Mobility Score^8^ is a functional assessment score for mobility. It assesses the patient’s level of ambulatory assistance required during three different scenarios (indoors, outdoors and during shopping). Each question has a maximum score of 3. The level of function is directly proportional to the score attained, with higher scores signifying better functional status.

The Harris Hip Score^9^ is a holistic scoring system assessing patients in four main domains, (i) pain, (ii) function, (iii) deformity, and (iv) range of motion. The total score is 100, with higher scores representing better outcomes.

The 36-items short form survey (SF-36) was conducted pre-operatively and during each consultation to measure the quality of life for the patients in eight different domains: physical functioning (PF), role physical (RP), bodily pain (BP), general health (GH), vitality (VT), social functioning (SF), role emotional (RE), and mental health (MH).

Radiological parameters collected included Centrum-Column-Diaphyseal Angle (CCD), Tip-Apex-Distance (TAD), varus/valgus deformity, loss of reduction, implant loosening, implant breakage, screw migration, screw cut-out, delayed union, non-union and peri-implant fractures.

Statistical analysis was performed with STATA 16.1. Categorical variables were presented as numbers and percentages. Values of continuous variables were presented as mean ± standard deviation (SD) or as median with interquartile range (IQR). Histogram and kurtosis were used to determine data normality. Chi-square test or Fisher’s exact test was used as appropriate to compare categorical variables. Student’s t-test or Wilcoxon test was used as appropriate to compare continuous variables in two groups. One-way ANOVA or Kruskal–Wallis test was used to compare continuous variables in more than two groups. A 2-tailed p-value of less than 0.05 was considered significant for all tests.

## Results

**Fig. 1:**
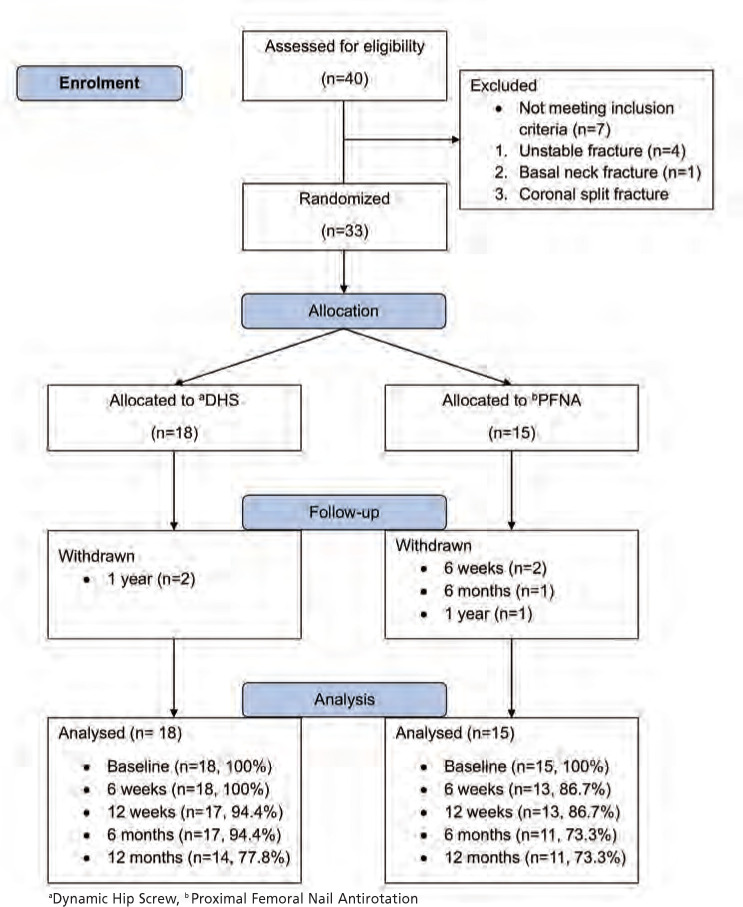
Consort Diagram.

Out of the 40 patients who presented with stable IT hip fractures during the study period, 33 patients met the inclusion criteria and were enrolled into the study. Seven patients were excluded after intra-operative imaging revealed unstable fracture patterns. The patient recruitment and participation in the study are presented in the CONSORT diagram (Fig. 1, CONSORT Diagram).

The study population demographics are shown in Table I. Both groups had similar demographic profiles (age, gender, body mass index, smoking status), pre-fall functional status (Parker mobility score) and quality of life (SF-36).

**Table I:** Comparison of patient demographics and fracture configuration between the dynamic hip screw (DHS) and the proximal femoral nail antirotation (PFNA II) groups.

	DHS (n=18)	PFNA (n=15)	p-value
**Age**	**Median (IQR^a^)**	**Median (IQR)**	0.11
	76.1 (72.3, 79.7)	70.9 (66.2, 78.7)	
**Gender**	**N (%)**	**N (%)**	0.99
Male	8 (44.4)	6 (40.0)	
Female	10 (55.6)	9 (60.0)	
**Ethnicity**	**N (%)**	**N (%)**	0.99
Chinese	14 (77.8)	13 (86.7)	
Malay	2 (11.1)	2 (13.3)	
Indian	1 (5.6)	0 (0.0)	
Others	1 (5.6)	0 (0.0)	
**BMI^b^**	**Median (IQR)**	**Median (IQR)**	0.88
	24.6 (20.0, 27.0)	23.3 (20.7, 26.3)	
**Smoking**	**N (%)**	**N (%)**	0.99
Non-smoker	16 (88.9)	14 (93.3)	
Ex-smoker	1 (5.6)	1 (6.7)	
Smoker	1 (5.6)	0 (0.0)	
**SF 36^c^**	**N (%)**	**N (%)**	0.94
Poor	0 (0.0)	0 (0.0)	
Fair	4 (23.5)	3 (21.4)	

Notes – ^a^ IQR: Interquartile range, ^b^ BMI: Body mass index, ^c^ SF 36: short form 36.

Surgical time for both groups was similar. The DHS group had a median surgical time of 47.5 minutes (IQR 40,60) and the PFNA II group had a median surgical time of 45 minutes (IQR 45,50) (p>0.05). Table II illustrates the intra-operative variables measured. We did not find any difference in terms of type of anaesthesia and estimated blood loss between the two implants. We also gathered data on technical problems during surgery, iatrogenic femur fractures as well as whether there were changes to the surgical procedure. None of the patients had the above intra-operative complications.

**Table II:** Comparison of per-operative findings between the dynamic hip screw (DHS) and the proximal femoral nail antirotation (PFNA II) groups.

	DHS (n=18)	PFNA (n=15)	p-value
**Type of Anaesthesia**	**N (%)**	**N (%)**	0.99
General Anaesthesia	4 (22.2)	3 (20.0)	
Regional Anaesthesia	14 (77.8)	12 (80.0)	
**Surgical Time (min)**	**Median (IQR^a^)**	**Median (IQR)**	0.70
	47.5 (40, 60)	45 (45, 55)	
**Blood Loss (ml)**	**N (%)**	**N (%)**	0.84
<50	9 (50)	9 (60)	
51-100	7 (38.9)	4 (26.7)	
101-150	1 (5.7)	0 (0.0)	
151-200	0 (0.0)	1 (6.7)	
201-250	1 (5.6)	1 (6.7)	
**Intra-operative complication**	**N (%)**	**N (%)**	-
Yes	0 (0.0)	0 (0.0)	
No	18 (100)	15 (100)	
**Intra-operative transfusion**	**N (%)**	**N (%)**	-
Yes	0 (0.0)	0 (0.0)	
No	18 (100)	15 (100)	
**Post-operative blood transfusion**	**N (%)**	**N (%)**	0.13
Yes	3 (16.7)	7 (46.7)	
No	15 (83.3)	8 (53.3)	

Note – ^a^ IQR: Interquartile range

No statistically significant difference was found between the two groups in terms of post-operative medical complications such as urinary tract infections, wound complications and wound infections (Table III). Post-surgery, there was also no significant difference in days to standing and days to ambulation between the two groups of patients. Functional assessment with the Parker Mobility Score and the Harris Hip Score for both groups did not reveal any statistically significant difference at all review intervals. We found a gradual improvement of both scores in the two groups as they progressed in their rehabilitation (Table IV).

**Table III:** Comparison of post-operative clinical and radiological parameters between the dynamic hip screw (DHS) and the proximal femoral nail antirotation (PFNA II) groups.

	DHS (n=18)	PFNA (n=15)	p-value
**Medical Complication**	**N (%)**	**N (%)**	0.99
Yes	1 (5.6)	1 (6.7)	
No	17 (94.4)	14 (93.3)	
**Wound Complication**	**N (%)**	**N (%)**	-
Yes	0 (0.0)	0 (0.0)	
No	18 (100)	15 (100)	
**Revision Surgery**	**N (%)**	**N (%)**	0.44
Yes	0 (0.0)	1 (9.1)	
No	14 (100)	10 (90.9)	
**Tip Apex Distance**	**N (%)**	**N (%)**	0.99
Acceptable	17 (94.4)	15 (100)	
Unacceptable	1 (5.6)	0 (18.2)	
**Varus-Valgus Deformity**	**N (%)**	**N (%)**	0.23
Present	3 (16.7)	0 (0.0)	
Absent	15 (83.3)	15 (100.0)	
**Loss of Reduction**	**N (%)**	**N (%)**	0.23
Present	3 (16.7)	0 (0.0)	
Absent	15 (83.3)	15 (100.0)	
**Fracture Impaction**	**N (%)**	**N (%)**	0.72
Present	8 (44.4)	5 (33.3)	
Absent	10 (55.6)	10 (66.7)	
**Periprosthetic Fracture**	**N (%)**	**N (%)**	-
Present	0 (0.0)	0 (0.0)	
Absent	18 (100)	15 (100)	
**Blade Perforation**	**N (%)**	**N (%)**	-
Present	0 (0.0)	0 (0.0)	
Absent	18 (100)	15 (100)	
**Malposition of Implant**	**N (%)**	**N (%)**	0.99
Present	1 (5.6)	1 (6.7)	
Absent	17 (94.4)	14 (93.3)	
**Implant Loosening**	**N (%)**	**N (%)**	-
Present	0 (0.0)	0 (0)	
Absent	18 (100)	15 (100)	
**Implant Failure**	**N (%)**	**N (%)**	0.46
Present	0 (0.0)	1 (6.7)	
Absent	18 (100)	14 (93.3)	

Note – ^a^ IQR: Interquartile range

**Table IV:** Comparison of post-operative functional score between the dynamic hip screw (DHS) and the proximal femoral nail antirotation (PFNA II) groups.

	DHS (n=18)	PFNA (n=15)	p-value
**Days to Standing (with Aid)**	**N (%)**	**N (%)**	0.99
1	13 (72.2)	12 (80.0)	
2	3 (16.7)	2 (13.3)	
3	2 (11.1)	1 (6.7)	
**Days to Ambulation (with Aid)**	**N (%)**	**N (%)**	0.21
1	8 (44.4)	6 (40.0)	
2	6 (33.3)	4 (26.7)	
3	4 (22.2)	1 (6.6)	
>3	0 (0.0)	4 (26.7)	
**Harris Hip Score**	**Median (IQRa)**	**Median (IQR)**	
6 Weeks	75 (67, 77)	73 (67, 80)	0.90
3 Months	76 (73, 81)	75.5 (68.5, 82.5)	0.66
6 Months	79 (72.5, 88.5)	84 (75, 91)	0.69
12 Months	84 (78, 95)	80 (76, 88)	0.20
**Parker Mobility Score**	**Median (IQR)**	**Median (IQR)**	
6 Weeks	5 (2.5, 6)	4 (2, 7)	0.99
3 Months	6 (4, 6)	6 (4, 8)	0.68
6 Months	7 (5, 9)	9 (4, 9)	0.83
12 Months	7.5 (7, 9)	7 (5, 9)	0.28
**Pain Score**	**Median (IQR)**	**Median (IQR)**	
At Discharge	2 (2,2)	2 (1, 2)	0.09
6 Weeks	2 (0, 4)	0 (0, 1)	0.20
3 Months	0 (0, 2)	0 (0, 0)	0.68
6 Months	0 (0, 0)	0 (0, 2)	0.19
12 Months	0 (0, 0)	0 (0, 1)	0.23

Note – ^a^ IQR: Interquartile range

Table III also shows that both groups had comparable postoperative radiological parameters such as tip apex distance (TAD), varus/valgus deformity and presence of fracture impaction on post-surgical and all follow-up radiographs. There were no periprosthetic fractures, implant cut outs or implant loosening in both groups throughout the study period.

One patient from each treatment arm had primary malposition of the implant. A patient who underwent DHS fixation had an initial TAD distance of 27mm and subsequently developed varus collapse with a CCD angle of 120° at the end of 1 year follow-up. The other patient who had undergone PFNA II fixation had primary posterior malpositioning of the blade and a TAD measurement of 25mm immediately post-surgery. Throughout the follow-up period, this patient did not develop any complications such as varus collapse or cut out. Both patients achieved fracture union by the end of the follow-up period.

**Fig. 2:**
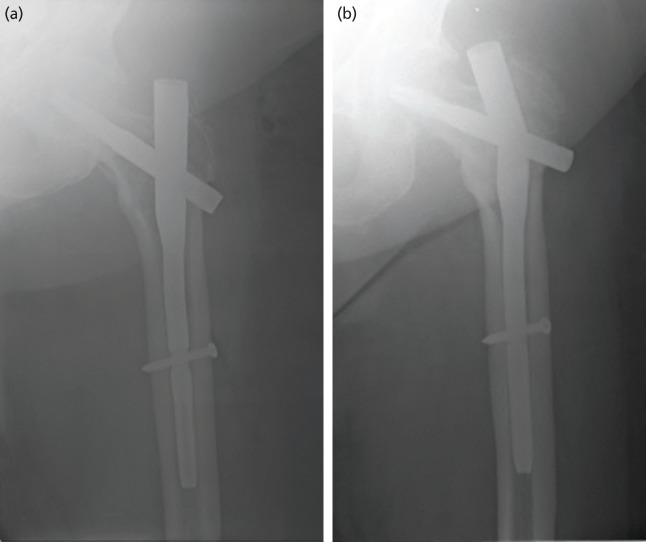
Case of Implant Failure. (a) Immediate post-op. (b) Four months post-op.

One patient who underwent PFNA II fixation developed an implant failure requiring revision surgery. Immediate postoperative radiograph showed that the CCD was 127° and TAD was 20mm. Intra-operative and post-operative recovery were uneventful for the patient. However, the patient experienced severe pain while carrying a heavy load 4 months after surgery. Plain radiograph and CT scan showed breakage of the PFNA II nail at the blade insertion site and that the IT fracture was not united. Fig. 2 shows the immediate post-operative and follow-up radiographs for this patient when the breakage occurred. The patient underwent revision surgery utilising the Trochanteric Fixation Nail Advance (Depuy Synthes TFNA) with cement augmentation and iliac crest bone grafting. The fracture subsequently united and the patient was able to ambulate with a walking stick at the end of the follow-up period.

## Discussion

Over the years, there has been much debate on the ideal implant for the treatment of IT fractures. A prospective randomised controlled trial by Reindl *et al* showed that the IM nail improved radiological outcomes with no added functional benefits to patients with unstable IT fractures^10^. However in a meta-analysis by Li *et al* on unstable IT fractures, the IM nail was shown to be superior to extramedullary fixations with better functional recovery and less blood loss^11^.

As expected, there is even greater controversy in the implant of choice for the management of stable IT fractures. Yu *et al* evaluated the use of the PFNA versus the DHS in stable IT fractures and found that the DHS had increased overall orthopaedic complications and re-operation rates^5^. However close to one thirds of the orthopaedic complications in the DHS group (n=10) was due to femur shaft fracture after implant removal, compared to three patients in the PFNA group. The author classified this complication separately from peri-implant fracture but did not specify the indications for implant removal and if the removal was performed routinely. We do not remove either implant routinely and if this complication is excluded from their results, their results mirrored ours with no difference between the two implants.

The use of PFNA II is not without its own set of complications. Blade cut out, nail fractures and distal shaft fractures were reported in the use of the PFNA^12^. In more recent years, many have noticed a “cut in” phenomenon associated with the PFNA as well^13^.

The incidence of cephalomedullary nail fracture is not frequent and ranges between 0.2-5.6%. Failure is associated with lower ASA scores, pathological fractures and subtrochanteric fractures^14,15^. In our study, we experienced one case of implant failure at four months post-operatively due to the PFNA II nail fracture at the blade insertion site. Radiological evaluation showed that the fracture had not fully united even though the patient was four months post-surgery. We postulate that delayed union resulted in fatigue of the implant with subsequent mechanical failure.

The use of IM nail in the fixation of unstable IT fracture resulted in earlier mobilisation and functional recovery in many studies^16-18^. These results however were not replicated in our study. We did not find any statistically significant difference between the two groups in terms of Harris Hip Scores (HHS) and Parker Mobility Scores for the entire study period. In stable hip fractures, reductions are typically performed closed using the traction table, and soft tissue dissection is often minimal. We postulate that this contributes to better pain control and faster rehabilitation, diminishing any potential advantages of the PFNA II over the DHS.

This contrasts with open reduction and the use of the trochanteric stabilisation plate that may be required for unstable IT fractures for which DHS fixation is used. This may cause more pain and slow down the functional recovery of these patients compared to the PFNA II group.

In a recent prospective randomised controlled trial by Singh *et al,* the authors concluded that functional outcomes with the DHS were comparable to the PFNA II in the management of stable IT fractures^19^. This is similar to what we found in our study. They had studied 60 elderly patients above the age of 60 with stable (31 A1.1e31 A2.1) intertrochanteric fractures. They had used Modified Harris Hip Score (MHHS), and Short form-12(SF-12) to score functional outcomes.

However, they found that the DHS group had almost double the bleeding (207ml) and longer surgical time (71.1 minutes) compared to the PFNA group. These findings were statistically significant. This may have likely been because they had used long incisions (10cm) for DHS fixation to accommodate 5 hole plates.

There are some other studies that have shown less soft tissue dissection, lower blood loss and shorter surgical time with the use of the PFNA II over the DHS^20,21^. This is likely due to comparatively smaller incisions with the PFNA II compared to the DHS especially when comparing longer DHS plates.

In our study, we found that there were no differences between both groups in terms of blood loss and surgical time. In fact, the median surgical time for the DHS group was 47.5 minutes and blood loss for 90% of the patients was less than 100ml even though tranexamic acid was not administered in our study population. Most patients in our study who had DHS fixation had two hole plates used. These findings show that the DHS with shorter plates can also be used safely in high-risk elderly patients with concerns of blood loss and anaesthetic time.

In our study, we observed similar intra-operative bleeding, total surgical time and post-operative pain scores in the two groups. The surgeries were conducted or supervised by fellowship trained orthopaedic trauma surgeons. We believe that once the surgeon has overcome the learning curve required for each implant, soft tissue dissection and surgical time would be comparable.

The cost of the PFNA II implant is substantially higher compared to the DHS implant. A short PFNA II implant costs USD$1220 compared to a 2-hole DHS at USD$490 in our institution. The cost difference is 250% and is justified in the treatment of unstable IT fracture as the PFNA has shown to have lower implant failures, lower revision rates and better post-surgical functional recovery^17^. However, in our study the use of the PFNA II did not provide these benefits in the treatment of stable IT fractures. Cost analysis studies on the treatment of IT fracture also showed that the DHS implant is more cost effective for stable IT fractures after taking into account total inpatient cost, revision surgery and quality of life^22,23^.

The strengths of our study include the rigorous adherence to our prospective protocol. All operations were conducted or supervised by a fellowship trained orthopaedic trauma consultant. Our patients were managed under an ortho-geriatric co-managed hip fracture care pathway and they followed a standardised post-operative rehabilitation protocol. Throughout the study period, we evaluated the participants with two functional scores, namely the Harris Hip Score and Parker Mobility Score. Additionally, we compared the number of days to standing and ambulation. These enabled us to provide a more comprehensive functional outcome assessment compared to other prospective randomised controlled trials^3,24^.

We acknowledge the limitations of our study. The study numbers remained small despite a long recruitment period. This was primarily due to a higher proportion of our elderly hip fracture patients having dementia and poor premorbid ambulatory status for which they had to be excluded. In addition, the difficulty in identifying suitable stable IT fractures for recruitment, perhaps as a factor of severe osteoporosis in our population, also hampered our recruitment. Finally, the duration of follow-up of one year in our study may not adequately capture long-term complications.

## Conclusion

Our study found that the DHS performed as well as the PFNA II for stable IT fractures in elderly patients. Both groups had similar intra-operative blood loss, surgical time and postoperative radiographic parameters. The DHS implant had non-inferior functional scores (Parker Mobility Scores and Harris Hip Scores) compared with the PFNA II. Studies with larger sample sizes can be undertaken to validate these results. We recommend the use of the DHS for this fracture type in view of its cost savings and equivalent outcomes.
